# The Fatty Liver Index’s Association with Incident Chronic Kidney Disease in Korean Middle-Aged Adults: A Community-Based Cohort Study

**DOI:** 10.3390/jcm13061616

**Published:** 2024-03-12

**Authors:** Hyun Hee Lee, Han Ro, Ji Yong Jung, Jae Hyun Chang, Wookyung Chung, Ae Jin Kim

**Affiliations:** 1 Division of Nephrology, Department of Internal Medicine, Gachon University Gil Medical Center, Incheon 21565, Republic of Korea; 2 Department of Internal Medicine, Gachon University College of Medicine, Incheon 21565, Republic of Korea

**Keywords:** fatty liver index, chronic kidney disease, estimated glomerular filtration rate, proteinuria

## Abstract

(1) **Background**: The relationship between nonalcoholic fatty liver disease (NAFLD) and incident chronic kidney disease (CKD) is unclear, and long-term follow-up data are limited. Therefore, this study aimed to evaluate whether NAFLD, as assessed by the fatty liver index (FLI), could predict the development of CKD in a community-based Korean cohort over 16 years. (2) **Methods**: Among the 10,030 total participants, 7778 patients without CKD were selected from the Korean Genome and Epidemiology Study (KoGES). The FLI grade ranged from 0 to 100 and was divided into three groups: low (FLI, <30), intermediate (FLI, 30–59), and high (FLI, ≥60). An estimated glomerular filtration rate (eGFR) of <60 mL/min/1.73 m^2^ or the development of proteinuria was considered to indicate incident CKD. (3) **Results**: During the 16-year follow-up period, 919 individuals (11.8%) developed CKD. The HRs of incident CKD in the intermediate FLI group (30–59) and high FLI group (≥60) increased compared with the reference low FLI group (<30) after adjusting for potentially confounding variables. NAFLD, as assessed by the FLI, was an independent risk factor for CKD. (4) **Conclusions**: Our findings suggest that the FLI, a simple surrogate biomarker of fatty liver disease, may be used to identify people at high risk of incident CKD in clinical practice.

## 1. Introduction

Chronic kidney disease (CKD) is a global health problem that affects approximately 8–18% of the adult population [1,2,3]. Its prevalence is expected to increase with an increase in metabolic risk factors, such as diabetes, obesity, and hypertension [1,4]. CKD causes significant morbidity and mortality and can progress to kidney failure with replacement therapy (KFRT). CKD is also an independent risk factor for cardiovascular disease, decreased quality of life, and cognitive impairment [5,6,7]. Therefore, there is increasing interest in identifying potentially modifiable risk factors for CKD in addition to conventional risk factors, such as diabetes and hypertension.

The most common cause of chronic liver disease is nonalcoholic fatty liver disease (NAFLD), which affects up to 25–30% of the general population [8,9]. NAFLD is defined by the presence of steatosis in more than 5% of hepatocytes without excessive alcohol intake or other causes of liver disease [10]. NAFLD encompasses a wide range of liver diseases, from simple steatosis to nonalcoholic steatohepatitis (NASH) and liver cirrhosis, followed by hepatocellular carcinoma [11,12]. Previous studies have shown that NAFLD causes progressive liver disease and diabetes, cardiovascular disease, and other severe extrahepatic complications [13]. Furthermore, recent evidence revealed a relationship between NAFLD and incident CKD [3,14,15,16,17,18]. However, there are some controversies about the relationship between NAFLD and CKD based on race, region, and other demographic characteristics [19,20,21].

The gold standard for fatty liver diagnosis is a liver biopsy, which is invasive and has a risk of complications [22]. Ultrasonography is the most commonly often used diagnostic test for fatty liver. However, this test has the limitation of having low sensitivity in mild cases of fatty change (less than 20–30%) and being dependent on the subjective assessment of operators [22]. According to Bedogni et al., the fatty liver index (FLI), a simple scoring system, predicts hepatic steatosis [23]. Previous studies have shown that the FLI correlates strongly with fatty liver when assessed by ultrasonography or proton magnetic resonance spectroscopy [24,25,26,27,28]. Therefore, this study aimed to evaluate whether NAFLD, as assessed by the FLI, was associated with incident CKD in a community-based Korean cohort. Over a 16-year follow-up period, we also investigated whether the FLI was an independent predictor of incident CKD, regardless of confounding factors such as diabetes mellitus, hypertension, and systemic inflammation.

## 2. Materials and Methods

### 2.1. Study Design and Participants

This study used data from the Ansan and Ansung cohort of the Korean Genome and Epidemiology Study (KoGES), a community-based, prospective cohort study conducted by the Korean Centers for Disease Control and Prevention [29]. This longitudinal cohort study examined genetic and environmental risk factors for chronic diseases. The design and survey methods of the KoGES have previously been described [29]. Briefly, the Ansan and Ansung cohort included men and women aged 40–69 years who were residents of two areas: an industrialized community located southwest of Seoul, Ansan, and a rural area in the south of Seoul, Ansung. Registered residents were randomly selected and contacted by mail, phone, or house visit in order to gather representative samples for statistically reliable findings. The respondents received an invitation to visit the hospital for a survey conducted by trained staff and a physical examination. The followed-up participants received an invitation via mail and telephone call to complete the surveys periodically. Between 2001 and 2002, 10,030 participants voluntarily participated in the baseline study (Ansan, *n* = 5012, response rate = 45.7%; Ansung, *n* = 5018, response rate = 69.6%). The cohort was surveyed every 2 years until 2019–2020. We analyzed data from 2001–2002 to 2017–2018. Kim et al. [29] reported that the comparison of health outcomes between the KoGES cohort, the Korea National Health and Nutrition Examination Survey (KNHANES III, 2005), and national cancer statistics (Korea Central Cancer Registry, KCCR) provides evidence to support the generalizability of the KoGES data to the Korean population.

**Figure 1:**
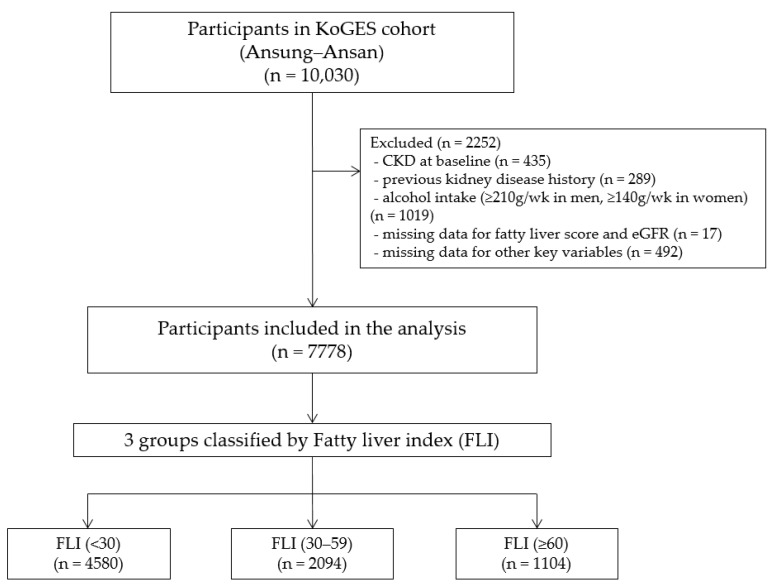
Flowchart of the study population. CKD, chronic kidney disease; eGFR, estimated glomerular filtration rate; FLI, fatty liver index; KoGES, Korean Genome and Epidemiology Study.

Among the 10,030 survey participants, 435 (4.3%) had previously been diagnosed with chronic kidney disease, 289 (2.9%) had a history of kidney disease, and 1019 (10.2%) had considerable alcohol intake (≥210 g/weeks in men and ≥140 g/weeks in women) and were excluded. Participants with missing FLI or estimated glomerular filtration rate (eGFR) data (*n* = 17) and other critical variables (*n* = 492) were also excluded. After these exclusions, the final analysis included 7778 participants (3383 men and 4395 women; Figure 1). All the participants voluntarily enrolled in the study and provided written informed consent. This study was conducted in accordance with the Declaration of Helsinki. The study protocol was approved by the Korea National Institute of Health Ethics Committee, the Korea Disease Control and Prevention Agency, and the Institute Review Board at the Gachon University Gil Medical Center (GCIRB2022-097; 6 April 2022).

### 2.2. Data Collection and Measurements

All the participants completed a self-administered standardized questionnaire on their demographic, behavioral, and socioeconomic demographics, such as age, sex, smoking status, alcohol intake, physical activity, income, education level, and medical history.

Trained healthcare providers also assessed anthropometric parameters using standard methods while the participants wore light-weight clothing without shoes. After the patients had relaxed for at least 10 min, the blood pressure (BP) was taken in the sitting position using a standard mercury sphygmomanometer (Baumanometer, Baum Co. Inc., Copiague, NY, USA). The height and weight were measured to the closest 0.1 cm and 0.1 kg. Their body mass index (BMI) was calculated as the body weight divided by the height squared (kg/m^2^). The body composition was determined using multifrequency bioelectrical impedance analysis (BIA; InBody 3.0, Biospace, Seoul, Republic of Korea).

Smoking status and alcohol intake were classified as never, former, or current. The education level was divided into three groups: low (lower than middle school), middle (middle school), and high (higher than middle school). Income was also divided into three groups: low (<USD 850 per month), middle (USD 850 to USD 2500 per month), and high (>USD 2500 per month).

Hypertension was defined as a medical history of hypertension, the use of antihypertensive drugs, or systolic BP (SBP) of ≥140 mmHg or diastolic BP (DBP) of ≥90 mmHg. Diabetes mellitus was defined as a medical history of diabetes mellitus, the use of antidiabetic drugs or insulin for hyperglycemia, a fasting glucose level of ≥126 mg/dL, a post-load glucose level of ≥200 mg/dL after a 75 g oral glucose tolerance test, or a hemoglobin A1c (HbA1c) of ≥6.5%. Cardiovascular disease was defined as a composite of myocardial infarction, congestive heart failure, coronary artery disease, cerebrovascular accident, or peripheral artery disease.

After an 8 h fast, blood and urine samples were collected and delivered to a central laboratory (Seoul Clinical Laboratories, Seoul, Republic of Korea) within 24 h. The serum concentrations of creatinine, blood urea nitrogen, albumin, aspartate aminotransferase (AST), alanine aminotransferase (ALT), gamma-glutamyl transferase (γ-GT), total cholesterol, high-density lipoprotein (HDL) cholesterol, and triglyceride (TG) were measured using a Hitachi Auto Analyzer 7600 (Hitachi, Tokyo, Japan). The hemoglobin (Hb) levels were measured using an ADVIA 120 hematology system (Bayer, Pittsburgh, PA, USA). The HbA1c levels were determined using high-performance liquid chromatography (Variant II; Rio-Rad Laboratories, Hercules, CA, USA). The high-sensitivity C-reactive protein (hs-CRP) levels were assessed using a turbidimetric immunoassay (ADVIA 1650 Auto Analyzer, Siemens, Tarrytown, NY, USA). The insulin concentrations in the blood were determined using a radioimmunoassay (Gamma Counter Cobra, Packard, Conroe, TX, USA). Insulin resistance was assessed using the homeostasis model assessment of insulin resistance (HOMA-IR) equation (fasting insulin [μIU/mL] × fasting glucose (mg/dL)/405) [30]. The eGFR was calculated using the CKD epidemiology collaboration (CKD-EPI) equation [31]. The urine test was performed semiquantitatively on fresh urine samples using a dipstick test (URISCAN Pro II; YD Diagnostics, Yongin-si, Republic of Korea). The urine protein levels are reported as one of six grades: negative, trace, 1+, 2+, 3+, or 4+. Proteinuria was defined as urine protein levels higher than trace levels. The laboratory parameters were measured at baseline and every 2 years thereafter.

### 2.3. Study Definitions and Outcomes

The FLI was calculated to determine the NAFLD status based on TG, γ-GT, BMI, and waist circumference (WC) measurements using the following formula:

$$\mathrm{FLI}=\frac{e^{0.953\times \ln\left(\mathrm{TG}\right)+0.139\times \mathrm{BMI}+0.178\times \ln\left(\gamma -\mathrm{GT}\right)+0.053\times \mathrm{WC}–15.745}}{{1+e}^{0.953\times \ln\left(\mathrm{TG}\right)+0.139\times \mathrm{BMI}+0.178\times \ln\left(\gamma -\mathrm{GT}\right)+0.053\times \mathrm{WC}–\;15.745}}\times \;100,$$

where TG is measured in mg/dL, γ-GT in U/L, WC in cm, and BMI in kg/m^2^ [23].

The participants were divided into three groups based on the FLI score: low (FLI, <30), intermediate (FLI, 30–59), and high (FLI, ≥60).

The primary outcome was incident CKD, defined as an eGFR of <60 mL/min/1.73 m^2^ or the development of proteinuria. The first of these measurements became the study endpoint. The study observation ended on 31 December 2018.

### 2.4. Statistical Analysis

Continuous data are expressed as the median and interquartile range (IQR). Categorical data are presented as frequencies and percentages. The FLI baseline characteristics were compared using a one-way analysis of variance or the Kruskal–Wallis test, depending on the normality of the continuous variable distributions. The chi-square test was used to compare categorical variables. A Kaplan–Meier curve was used to determine the cumulative incidence of CKD. The differences in the cumulative incidence of CKD among the groups were determined using log-rank tests. The independent relationship of the FLI status with incident CKD was determined using the Cox proportional hazard regression model. The covariates were adjusted for four different models. Model 1 was adjusted for age and sex. Model 2 was further adjusted for socioeconomic factors and social behaviors, including education, income level, alcohol intake, and smoking habits. Model 3 was further adjusted for the factors above and the laboratory results, such as eGFR, albumin, Hb, hs-CRP, and HOMA-IR. Model 4 was fully adjusted for the factors above and comorbidities, including hypertension, diabetes mellitus, and cardiovascular disease. The evaluation of the possible nonlinear relationship between the FLI and the hazard ratio (HR) of CKD was performed using restricted cubic splines with the total adjustment (model 4) of confounding factors.

We also conducted subgroup analyses by age (<60 and ≥60 year), sex, education, income, alcohol intake, smoking, hypertension, diabetes mellitus, and cardiovascular disease. The interaction terms between each stratifying factor and the FLI on incident CKD were assessed in adjusted Cox proportional hazard models using the likelihood ratio test for the subgroup analysis. The fully adjusted model 4 of the multivariable Cox proportional hazard models was used to compare FLI groups 2 and 3 with group 1. All the analyses were conducted using SPSS version 22.0 (SPSS Inc., Chicago, IL, USA) or R version 4.2.0 (The R Foundation for Statistical Computing, Vienna, Austria). All the statistical tests were two-sided; *p* values < 0.05 were considered statistically significant.

## 3. Results

### 3.1. Baseline Characteristics

The baseline characteristics of the participants based on their FLI scores are shown in Table 1. This study included a total of 7778 participants. FLI < 30, FLI 30–59, and FLI ≥ 60 had median ages of 49.0, 52.0, and 51.0 year, respectively. Men were more prevalent in the higher FLI grades (35.8%, 50.0%, and 63.2% for FLI < 30, FLI 30–59, and FLI ≥ 60, respectively).

The high FLI grade had higher median values for BMI, WC, SBP, DBP, Hb, albumin, AST, ALT, γ-GT, total cholesterol, TG, Hb A1c, and HOMA-IR than the low FLI grade. In addition, various hepatic function indicators, such as AST and ALT, increased with the higher FLI grade. The proportions of current alcohol intake and smoking increased with the FLI grade. Moreover, compared with the FLI < 30 group, the FLI 30–59 and FLI ≥ 60 groups had higher rates of current alcohol intake (*n* = 1741, 38.0% in FLI < 30; *n* = 930, 44.4% in FLI 30–59; *n* = 609, 55.2% in FLI ≥ 60) and smoking (*n* = 851, 18.6% in FLI < 30; *n* = 539, 25.7% in FLI 30–59; *n* = 370, 33.5% in FLI ≥ 60) and higher prevalence of hypertension (*n* = 1196, 26.1% in FLI < 30; *n* = 979, 46.8% in FLI 30–59; *n* = 637, 57.7% in FLI ≥ 60), diabetes mellitus (*n* = 268, 5.9% in FLI < 30; *n* = 300, 14.3% in FLI 30–59; *n* = 270, 24.5% in FLI ≥ 60), and cardiovascular disease (*n* = 109, 2.4% in FLI < 30; *n* = 73, 3.5% in FLI 30–59; *n* = 36, 3.3% in FLI ≥ 60).

### 3.2. Relationship between FLI and Kidney Function

#### 3.2.1. Incidence of CKD

Table 2 shows the incidence of CKD across the 16-year follow-up. During this period, 919 (11.8%) of the 7778 participants were newly diagnosed with CKD.

#### 3.2.2. FLI as a Predictor of CKD

**Figure 2:**
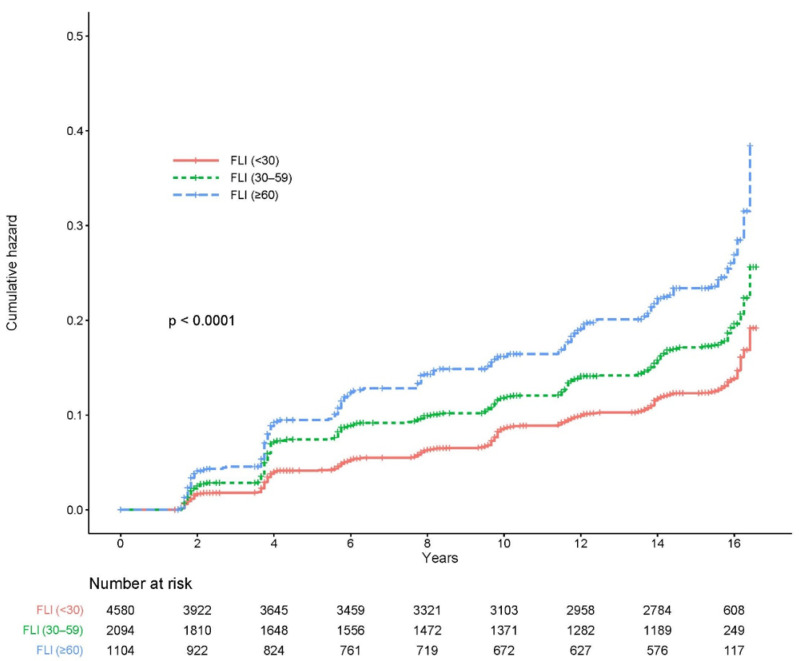
Cumulative incidence of CKD based on the FLI group. CKD, chronic kidney disease; FLI, fatty liver index.

Figure 2 shows the cumulative incidence of CKD according to the FLI stage as a Kaplan–Meier curve. The cumulative incidence of CKD over 16 years significantly increased as the FLI grade increased (log-rank test *p* < 0.0001).

**Figure 3:**
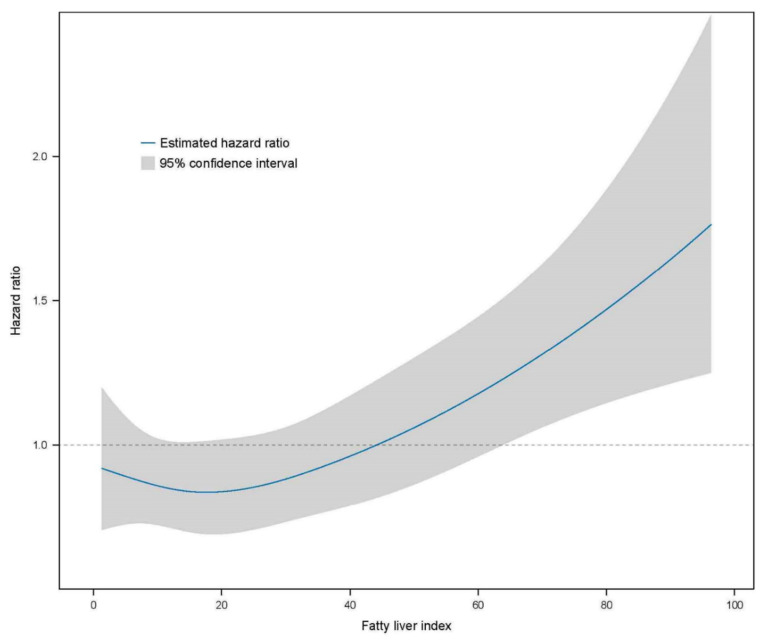
Restricted cubic spline curve for the risk of incident CKD by the FLI. The FLI had a linear relationship with the risk of incident CKD. CKD, chronic kidney disease; FLI, fatty liver index.

Further analyses were performed using multivariate Cox proportional hazard regression to predict CKD based on the FLI stage (Table 3). The HR of incident CKD increased in the higher FLI group compared with the FLI < 30 group (reference). The HRs of the CKD incidence in the FLI 30–59 group were 1.396 (95% CI, 1.202–1.621) in the crude analysis, 1.258 (95% CI, 1.083–1.462) in model 1, 1.261 (95% CI, 1.085–1.465) in model 2, 1.236 (95% CI, 1.059–1.444) in model 3, and 1.171 (95% CI, 1.000–1.371) in model 4 compared with the reference FLI < 30 group. The HRs of the CKD incidence for FLI ≥ 60 were 1.945 (95% CI, 1.643–2.303) in the crude analysis, 1.832 (95% CI, 1.544–2.173) in model 1, 1.879 (95% CI, 1.582–2.232) in model 2, 1.726 (1.443–2.065) in model 3, and 1.594 (1.325–1.918) in model 4 compared with the reference FLI < 30 group. The restricted cubic spline curve indicated a graded increase in the HRs after total adjustment for confounding factors based on the FLI (Figure 3).

### 3.3. Subgroup Analyses

**Figure 4:**
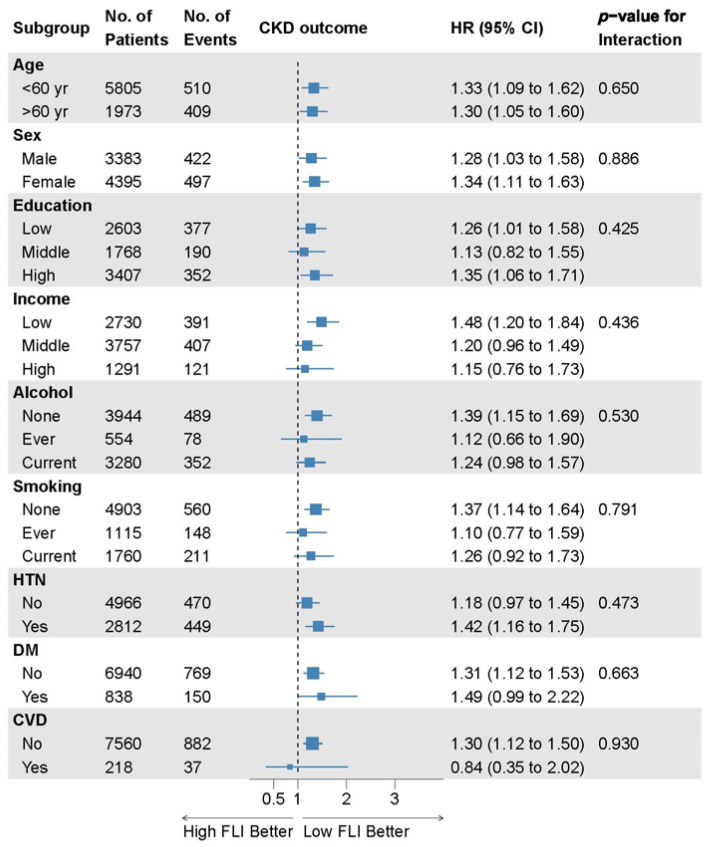
Subgroup analyses of the relationship between the FLI and incident CKD. Data are collected from the Cox proportional hazard analysis with covariate adjustment model 4 comparing FLI groups 2 and 3 to group 1. Here, 95% CI, 95% confidence interval; CKD, chronic kidney disease; CVD, cardiovascular disease; DM, diabetes mellitus; FLI, fatty liver index; HR, hazard ratio; HTN, hypertension; No., number.

There were no significant interactions between the FLI and incident CKD subgroups in the prespecified subgroup analyses (Figure 4).

## 4. Discussion

In this large-scale longitudinal cohort study, we found that a higher FLI was associated with an increased risk of incident CKD throughout the 16-year follow-up period. This association was consistent after adjusting for traditional risk factors, such as diabetes, hypertension, and systemic inflammation, in specified subgroup analyses. To the best of our knowledge, this is the longest follow-up prospective cohort study to investigate the effect of the FLI on incident CKD risk prediction.

Previous studies have shown that patients with NAFLD have an increased risk of developing CKD. However, whether NAFLD is an independent risk factor for incident CKD remains controversial. Sinn et al. [32] found that NAFLD diagnosed using ultrasonography was associated with an increased risk of developing CKD after a median follow-up of 4.15 years in 41,430 patients who underwent repeated health check-ups. However, ultrasonography is prone to measurement inaccuracy. Because of the long duration of the study, multiple radiologists performed the abdominal ultrasonography, which increased the measurement variability. Huh et al. [3] showed that NAFLD assessed by the FLI is an independent risk factor for incident CKD in a community-based cohort of 4761 people during a 10-year period. However, proteinuria was not included in determining the outcome of incident CKD. On the other hand, a secondary analysis of the Framingham Heart Study showed no significant relationship between liver fat assessed by attenuation on CT and prevalence or incidence over a median follow-up of 12.5 years [33]. They suggested that the relationship between NAFLD and CKD is explained by shared risk factors. This finding is consistent with the German population-based KORA cohort study, which found an attenuated association between fatty liver estimated by the FLI and CKD development after adjusting for cardiometabolic risk factors [18]. Therefore, we evaluated whether NAFLD predicts incident CKD in Korean middle-aged adults, independent of other confounding cardiometabolic risk factors.

The FLI was found to be a significant predictor of incident CKD. Because fatty liver disease is becoming more common, it is crucial to identify high-risk individuals early to determine whether they require further imaging tests, such as magnetic resonance spectroscopy and ultrasound. Although ultrasonography is commonly used to diagnose fatty liver disease, it is insensitive to mild steatosis [34]. Computed tomography (CT) and magnetic resonance imaging (MRI) have higher sensitivity for assessing fatty liver. However, the potential radiation risks of CT and the high cost of MRI prevent them from being widely available tools [22,28]. Although liver biopsy is the gold standard for diagnosing NAFLD, its invasive nature, high cost, and sampling inaccuracy make it unsuitable for frequent use in clinical practice [22]. However, the FLI is a simple index and a cost-effective tool because it only requires the BMI, WC, γ-GT, and TG. The FLI may diagnose fatty liver disease as indicated by ultrasonography [23] and magnetic resonance spectroscopy [28] with high accuracy and has been well-validated in Korean and other populations [24,25,27,35]. These advantages make the FLI a valuable marker for population studies. Even after adjusting for age, cardiometabolic risk, and inflammation, the presence of NAFLD assessed by the FLI was associated with incident CKD in this study. Therefore, the FLI is a simple and valuable surrogate marker for NAFLD and incident CKD.

Although the pathophysiological mechanisms behind the development of CKD in NAFLD are unknown, various plausible mechanisms have been presented. The liver is a primary source of inflammatory cytokines and a critical glucose and lipid metabolism regulator [36]. Systemic release of various proinflammatory, profibrogenic, pro-oxidant, and procoagulant mediators, such as interleukin-6 (IL-6), tumor necrosis factor-α (TNF-α), transforming growth factor-β (TGF-β), C-reactive protein (CRP), plasminogen activator inhibitor-1 (PAI-1), and fibrinogen, in steatotic and inflamed livers can contribute to CKD development [37,38,39]. The activation of the renin–angiotensin–aldosterone system (RAAS) is also suggested to have a role in the pathogenesis of NAFLD and CKD [36,40]. Angiotensin II causes insulin resistance, de novo lipogenesis, mitochondrial dysfunction, and the production of reactive oxygen species (ROS) and proinflammatory cytokines in the liver, which leads to fibrogenesis [41]. RAAS activation in the kidney increases renal ectopic lipid deposition, which causes oxidative stress and inflammation via hemodynamic and nonhemodynamic effects, leading to glomerulosclerosis, tubulointerstitial inflammation, and fibrosis [36,42]. Increased systemic and hepatic insulin resistance and the development of atherogenic dyslipidemia are associated with renal endothelial dysfunction, renovascular damage, and glomerular injury in NAFLD patients, contributing to CKD development [43]. Furthermore, recent evidence has shown that intestinal microbiota dysbiosis in NAFLD patients increases intestinal permeability, secondary bile acids, and uremic toxins, leading to renal damage [44].

This study has some limitations. First, although the FLI had previously been validated as a marker for NAFLD, this cohort study did not include imaging studies, such as ultrasounds and magnetic resonance spectroscopy or histologic data, which are the gold standard for diagnosing NAFLD. Second, we used dipstick urinalysis to diagnose proteinuria. Only a subset of the cohort had spot urine albumin-to-creatinine test results. Although it is the gold standard for quantitating proteinuria, 24 h urine collection is complex to perform consistently in a large epidemiologic study. Third, we could not obtain complete data, such as particular names or classifications of hypertension, diabetes, or other drugs that may impair renal function. Although we adjusted for multiple confounding factors, there is still a possibility that some unmeasured confounders could affect the relationship between NAFLD and CKD. Fourth, because our participants were drawn from the community-based Korean cohort, it is unclear whether our results could be applied to other regions and ethnic groups. Fifth, the study group was not a statistically representative random sample that accurately reflects the entire population, which is a common issue in many prospective cohort studies. Although this may not significantly affect the identification of associations between exposure and outcomes, it needs to be taken into account when applying the study findings to the entire population. Finally, because our study was observational, causality cannot be derived from our study. Despite these limitations, the present study is the longest follow-up study in a large population cohort indicating a relationship between NAFLD and CKD. For the definition of incident CKD, we used renal function with eGFR using creatinine and included proteinuria.

## 5. Conclusions

In conclusion, our study is the longest follow-up cohort study to examine the relationship between the FLI and incident CKD in a middle-aged adult population. Our findings show that the FLI is a simple and effective method for identifying people at high risk of CKD. In addition, NAFLD leads to the development of incident CKD independent of hypertension, diabetes, systemic insulin resistance, and inflammatory status. Further studies are needed to understand the mechanism underlying fatty liver disease’s independent role in incident CKD.

## Figures and Tables

**Table 1:** Baseline characteristics according to the FLI.

Variables	FLI			*p*
	<30	30–59	≥60	
Participants (*n*)	4580	2094	1104	
Age (yr)	49.0 (44.0–59.0)	52.0 (45.0–61.0)	51.0 (45.0–59.0)	<0.001
Sex (male), *n* (%)	1638 (35.8)	1047 (50.0)	698 (63.2)	<0.001
BMI (kg/m^2^)	23.1 (21.4–24.6)	25.9 (24.5–27.5)	27.9 (25.9–29.9)	<0.001
Waist circumference (cm)	77.3 (72.8–82.0)	87.0 (83.2–91.0)	92.0 (88.0–96.7)	<0.001
Education				<0.001
Low	1459 (31.9)	778 (37.2)	366 (33.2)	
Middle	1076 (23.5)	459 (21.9)	233 (21.1)	
High	2045 (44.7)	857 (40.9)	505 (45.7)	
Income				0.016
Low	1547 (33.8)	790 (37.7)	393 (35.6)	
Middle	2280 (49.8)	954 (45.6)	523 (47.4)	
High	753 (16.4)	350 (16.7)	188 (17.0)	
Alcohol drinking status, *n* (%)				<0.001
Never	2546 (55.6)	995 (47.5)	403 (36.5)	
Former	293 (6.4)	169 (8.1)	92 (8.3)	
Current	1741 (38.0)	930 (44.4)	609 (55.2)	
Smoking status, *n* (%)				<0.001
Never	3193 (69.7)	1213 (57.9)	497 (45.0)	
Former	536 (11.7)	342 (16.3)	237 (21.5)	
Current	851 (18.6)	539 (25.7)	370 (33.5)	
SBP (mmHg)	118.0 (108.0–130.0)	126.0 (114.0–138.0)	130.0 (120.0–142.0)	<0.001
DBP (mmHg)	78.0 (70.0–86.0)	82.0 (76.0–90.0)	88.0 (80.0–94.0)	<0.001
Creatinine (mg/dL)	0.8 (0.6–0.9)	0.8 (0.7–1.0)	0.9 (0.7–1.0)	<0.001
eGFR (mL/min/1.73 m^2^)	98.0 (85.0–107.2)	93.8 (81.5–102.9)	93.7 (80.6–103.4)	<0.001
Hemoglobin (g/dL)	13.1 (12.2–14.1)	13.9 (12.8–15.0)	14.5 (13.3–15.5)	<0.001
Albumin (g/dL)	4.1 (4.0–4.4)	4.2 (4.1–4.4)	4.2 (4.1–4.4)	<0.001
hs-CRP (mg/L)	0.1 (0.0–0.2)	0.2 (0.1–0.3)	0.2 (0.1–0.3)	<0.001
AST (U/L)	25.0 (22.0–29.0)	27.0 (23.0–32.0)	31.0 (26.0–39.0)	<0.001
ALT (U/L)	19.0 (16.0–25.0)	26.0 (20.0–34.0)	35.0 (26.0–50.0)	<0.001
γ-GT (U/L)	13.0 (10.0–19.0)	24.0 (16.0–40.0)	48.0 (29.0–81.0)	<0.001
Total cholesterol (mg/dL)	182.0 (161.0–206.0)	195.0 (174.0–219.0)	204.0 (180.0–230.0)	<0.001
HDL cholesterol (mg/dL)	46.0 (40.0–52.0)	40.0 (36.0–47.0)	39.0 (34.0–44.0)	<0.001
Triglyceride (mg/dL)	108.0 (86.0–138.0)	163.0 (130.0–215.0)	235.0 (177.0–320.0)	<0.001
HbA1c (%)	5.5 (5.3–5.7)	5.7 (5.4–6.0)	5.8 (5.5–6.3)	<0.001
HOMA-IR	1.3 (1.0–1.8)	1.6 (1.2–2.2)	2.0 (1.3–2.8)	<0.001
HTN, *n* (%)	1196 (26.1)	979 (46.8)	637 (57.7)	<0.001
DM, *n* (%)	268 (5.9%)	300 (14.3)	270 (24.5)	<0.001
Cardiovascular disease, *n* (%)	109 (2.4)	73 (3.5)	36 (3.3)	0.024

Data are expressed as the median (interquartile range) or number (percentage) and compared by one-way analysis of variance, Kruskal–Wallis test, or chi-square test. Abbreviations: γ-GT, gamma-glutamyl transferase; ALT, alanine aminotransferase; AST, aspartate aminotransferase; BMI, body mass index; DBP, diastolic blood pressure; DM, diabetes mellitus; eGFR, estimated glomerular filtration rate based on creatinine; FLI, fatty liver index; HbA1c, hemoglobin A1c; HDL, high-density lipoprotein; HOMA-IR, homeostasis model assessment of insulin resistance; hs-CRP, high-sensitivity C-reactive protein; HTN, hypertension; SBP, systolic blood pressure.

**Table 2:** Incidence of CKD during the follow-up study.

	FLI		
	<30	30–59	≥60
*n*	4580	2094	1104
Incident CKD (*n*)	447	279	193
Mean follow-up (yr)	11.1	10.8	10.1
Person-years of follow-up	50,688	22,587	11,191
Incidence rate per 1000 person-years	8.82	12.35	17.25

CKD, chronic kidney disease; FLI, fatty liver index.

**Table 3:** Relationship between the FLI and the risk of CKD.

	FLI			*p*
	<30	30–59	≥60	
Crude	1.00 (reference)	1.396 (1.202–1.621)	1.945 (1.643–2.303)	<0.001
Model 1	1.00 (reference)	1.258 (1.083–1.462)	1.832 (1.544–2.173)	<0.001
Model 2	1.00 (reference)	1.261 (1.085–1.465)	1.879 (1.582–2.232)	<0.001
Model 3	1.00 (reference)	1.236 (1.059–1.444)	1.726 (1.443–2.065)	<0.001
Model 4	1.00 (reference)	1.171 (1.000–1.371)	1.594 (1.325–1.918)	<0.001

Model 1: adjusted for age and sex. Model 2: adjusted for model 1 + education, income, alcohol intake, and smoking. Model 3: adjusted for model 2 + eGFR, albumin, hs-CRP, hemoglobin, and HOMA-IR. Model 4: adjusted for model 3 + hypertension, diabetes mellitus, and cardiovascular disease. CKD, chronic kidney disease; eGFR, estimated glomerular filtration rate; FLI, fatty liver index; HOMA-IR, homeostasis model assessment of insulin resistance; hs-CRP, high-sensitivity C-reactive protein.

## Data Availability

The data are available [http://www.cdc.go.kr, accessed on 19 April 2022] with the permission of the Korea Disease Control and Preventing Agency.
